# Impact of Phlorotannin Extracts from *Fucus vesiculosus* on Human Gut Microbiota

**DOI:** 10.3390/md19070375

**Published:** 2021-06-29

**Authors:** Marcelo D. Catarino, Catarina Marçal, Teresa Bonifácio-Lopes, Débora Campos, Nuno Mateus, Artur M. S. Silva, Maria Manuela Pintado, Susana M. Cardoso

**Affiliations:** 1 LAQV-REQUIMTE, Department of Chemistry, University of Aveiro, 3810-193 Aveiro, Portugal; mcatarino@ua.pt (M.D.C.); catarina.marcal@ua.pt (C.M.); artur.silva@ua.pt (A.M.S.S.); 2 CBQF-Centro de Biotecnologia e Química Fina–Laboratório Associado, Escola Superior de Biotecnologia, Universidade Católica Portuguesa, Rua Diogo Botelho 1327, 4169-005 Porto, Portugal; mvlopes@portu.ucp.pt (T.B.-L.); dcampos@porto.ucp.pt (D.C.); mpintado@porto.ucp.pt (M.M.P.); 3 REQUIMTE/LAQV, Department of Chemistry and Biochemistry, Faculty of Sciences, University of Porto, 4169-007 Porto, Portugal; nbmateus@fc.up.pt

**Keywords:** phlorotannins, brown seaweeds, gut microbiota, bioaccessibility, short-chain fatty acids, prebiotics, gastrointestinal tract

## Abstract

Recent studies indicate that plant polyphenols could be pointed as potential prebiotic candidates since they may interact with the gut microbiota, stimulating its growth and the production of metabolites. However, little is known about the fate of brown seaweeds’ phlorotannins during their passage throughout the gastrointestinal tract. This work aimed to evaluate the stability and bioaccessibility of *Fucus vesiculosus* phlorotannins after being submitted to a simulated digestive process, as well as their possible modulatory effects on gut microbiota and short-chain fatty acids production following a fermentation procedure using fecal inoculates to mimic the conditions of the large intestine. The stability of phlorotannins throughout the gastrointestinal tract was reduced, with a bioaccessibility index between 2 and 14%. Moreover, slight alterations in the growth of certain commensal bacteria were noticed, with *Enterococcus* spp. being the most enhanced group. Likewise, *F. vesiculosus* phlorotannins displayed striking capacity to enhance the levels of propionate and butyrate, which are two important short-chain fatty acids known for their role in intestinal homeostasis. In summary, this work provides valuable information regarding the behavior of *F. vesiculosus* phlorotannins along the gastrointestinal tract, presenting clear evidence that these compounds can positively contribute to the maintenance of a healthy gastrointestinal condition.

## 1. Introduction

The human intestinal tract harbors a complex community of microorganisms, collectively termed as intestinal or gut microbiota. The microbial colonization of the gastrointestinal tract starts right after birth and undergoes a symbiotic co-evolution along with their host, importantly contributing to the maintenance of intestinal homeostasis, development and integrity of the mucosal barrier, production of various nutrients, protection against microbial pathogens, maturation of the immune system and many other functions [1]. Throughout adulthood, the intestinal microbiota is regarded as relatively stable, although it may be affected by several extrinsic factors including dietary habits, medication (especially with antibiotics), environmental pollution and exposure to xenobiotics, physical activity and hygiene [2]. When these factors cause significant changes in the composition and/or function of the gut microbiota, the whole microbial ecosystem is perturbed to an extent that exceeds its resistance and resilience capabilities, leading to a condition known as dysbiosis. Consequently, dysbiosis has been associated with an increasing list of diseases, which include inflammatory bowel disease (IBD), irritable bowel syndrome (IBS), coeliac disease and colorectal cancer (CRC) [3]. Additionally, several extra-intestinal disorders such as asthma [4], systemic lupus erythematosus [5], cardiovascular disease [6] or even mental and neurodegenerative diseases including autism, anxiety, depression, chronic pain, Parkinson’s, Alzheimer’s or Huntington’s can be linked to a dysfunctional gut microbiota [7,8,9].

Based on this evidence, it is clear that the manipulation of gut microbiota could be regarded as a promising strategy to treat disease and improve health. In this context, prebiotics appear as important tools capable of manipulating and modifying the gut microbiota composition and promoting the host’s health status. They were first described in 1995 [10] and are currently defined as “a substrate that is selectively utilized by host microorganisms conferring a health benefit” [11]. In other terms, prebiotics are non-digestible dietary components that act as substrates that selectively stimulate the growth and/or biological activity of health-promoting bacteria residing in the host’s colon. Common prebiotics include several non-digestible polysaccharides, such as resistant starch and pectin, as well as oligosaccharides such as fructo-oligosaccharides (FOSs), galacto-oligosaccharides (GOSs), lactulose and inulin, which are found mainly in several land-vegetables, fruits and milk [12]. More recently, increasing evidence has shown that other compounds such as polyphenols and polyunsaturated fatty acids may also display modulatory effects on gut microbiota populations through selective prebiotic effects and antimicrobial activities against gut pathogenic bacteria [13,14]. Indeed, animal studies have demonstrated that the consumption of polyphenols, especially catechins, anthocyanins and proanthocyanidins, not only favors the growth of probiotic bacteria, such as *Lactobacillus*, *Bifidobacterium*, *Akkermansia*, *Roseburia* and *Faecalibacterium* spp., but also increases the production of short-chain fatty acids (SCFAs), including butyrate, which is the major energy source for the colonic epithelium and profoundly influences intestinal homeostasis [15]. Likewise, clinical trials have also revealed that the consumption of anthocyanins and ellagic acid promotes increases in *Lactobacillus acidophilus*, *Bifidobacterium* and *Faecalibacterium* spp. abundance in the stool and a reduction of the lipopolysaccharide-binding protein in the plasma of volunteers [16,17].

In contrast, studies regarding the prebiotic potential of seaweeds (particularly brown) are still scarce and essentially focused on the in vitro effects of their polysaccharides, while the fate of phlorotannins when crossing the gastrointestinal tract, remains deeply unexplored subject [18]. These compounds are specific phenolics biosynthesized only by brown seaweeds consisting of polymeric structures composed of several phloroglucinol units [18], and, despite the fact that they have been reported in the literature for their promising and versatile bioactive health benefits, only a limited number of studies have addressed their behavior in the gastrointestinal tract. Recent works have shown that an *Ecklonia radiata* phlorotannin-enriched extract performed better than inulin as it promoted a higher increase in *Lactobacillus*, *F. prausnitzii*, *C. coccoides*, Firmicutes and *E. coli* in fermentations conducted with human fecal microbiota [19], while the administration of *Lessonia turbeculata* polyphenol-rich extract to streptozotocin-induced diabetic rats was found to significantly restore the relative abundance of the overall bacterial diversity and SCFAs to levels similar to the negative control [20]. Interestingly, to the authors knowledge, no studies addressing the stability and bioactivity of phlorotannins throughout the gastrointestinal tract has been performed yet.

In this context, the aim of this work was to evaluate the stability and bioaccessibility of *F. vesiculosus* phlorotannin-rich extracts when crossing the gastrointestinal tract and ultimately disclose their possible modulatory effects toward the gut microbiota and short-chain fatty acid production.

## 2. Results and Discussion

### 2.1. Stability, Bioaccessibility and Antioxidant Activity of F. vesiculosus Extracts throughout the Simulated GIT

To evaluate the stability of *F. vesiculosus* phlorotannins throughout the digestive tract, both crude (CRD) and ethyl acetate fraction (EtOAc) were submitted to a simulated gastrointestinal (GIT) digestion and evaluated for their total phlorotannin content and antioxidant activity after each gut compartment. The results presented in Table 1 clearly demonstrate that the total phlorotannin content of the EtOAc fraction progressively decreased after each step from the GIT simulation. Interestingly, in the case of CRD, after the initial decrease in the mouth, an increase in the phlorotannin levels was found after the stomach digestion, followed by another decrease in the intestine. The reduction in the total phlorotannin content (TPhC) of the samples after the mouth digestion could be explained by possible interactions occurring between phlorotannins and the salivary proteins. In fact, such interactions are very well described for plant tannins and very relevant for the development of important sensory characteristics of certain foods and beverages such as wine [21]. The extreme pH conditions in the stomach can also explain why the TPhC of the EtOAc kept decreasing in this compartment. However, in the case of CRD, because this sample is more complex and contains other non-phlorotannin compounds, it is possible that such compounds might be interacting with phlorotannins, protecting them from reacting with the mouth proteins and degrading with the low stomach pH. In turn, the stomach pH may also promote the degradation of those non-phlorotannin compounds, promoting the release of the phlorotannins, making them more available to react with the 2,4-dimethoxybenzaldehyde (DMBA). In fact, similar observations have been previously reported for plant phenolics [22,23] and are on the basis of the delivery strategies in which phenolic compounds are encapsulated in order to resist the gastrointestinal conditions and reach intact for absorption in the intestines [24]. Additionally, it was noticed that even though undigested EtOAc had higher TPhC compared to the undigested CRD, after the stomach and intestine digestion, the TPhC of the latter was slightly higher compared with the EtOAc, which is in agreement with the hypothesis that the EtOAc phlorotannins were more exposed to the GIT degradation than those of CRD.

At the end of the simulated GIT, only a small portion of the total phlorotannins loaded in the system was bioaccessible, which is in line with previous studies carried out on land plant tannins. At this point, it is important to clarify that the term “bioavailability” expresses the fraction of an ingested compound/nutrient that reaches the systemic circulation to be distributed to organs and tissues and to manifest its bioactivity. However, before becoming bioavailable, the target compound/nutrient must be released from the food matrix and made available for bloodstream absorption, which is what defines the term “bioaccessibility” [25]. Interestingly, despite the fact that the undigested CRD exhibited lower TPhC compared to the undigested EtOAc, the bioaccessibility index of the former was 14.1%, while the latter was only 2.0%. Once again, this outcome might be in part explained by the fact that EtOAc experienced higher phlorotannin degradation than CRD, and, therefore, when the compounds reach the intestine to be absorbed, the TPhC of the matrix is already lower.

Concerning the antioxidant activity of the samples after each step of the GIT simulation, both NO^●^ and SO^●–^ results were in line with the TPhC of the respective samples, i.e., the samples with higher phlorotannin concentrations exhibited the lowest IC_50_ values and vice versa. Indeed, strong negative correlations were found between the TPhC and the antioxidant assays with CRD showing R^2^ of −0.82 and −0.94 and EtOAc showing R^2^ of −0.91 and −0.82 in NO^●^ and SO^●–^, respectively, thus indicating a clear association between the phlorotannin content after each step of the simulated digestion and the antioxidant activities observed.

### 2.2. Prebiotic Effect

**Figure 1:**
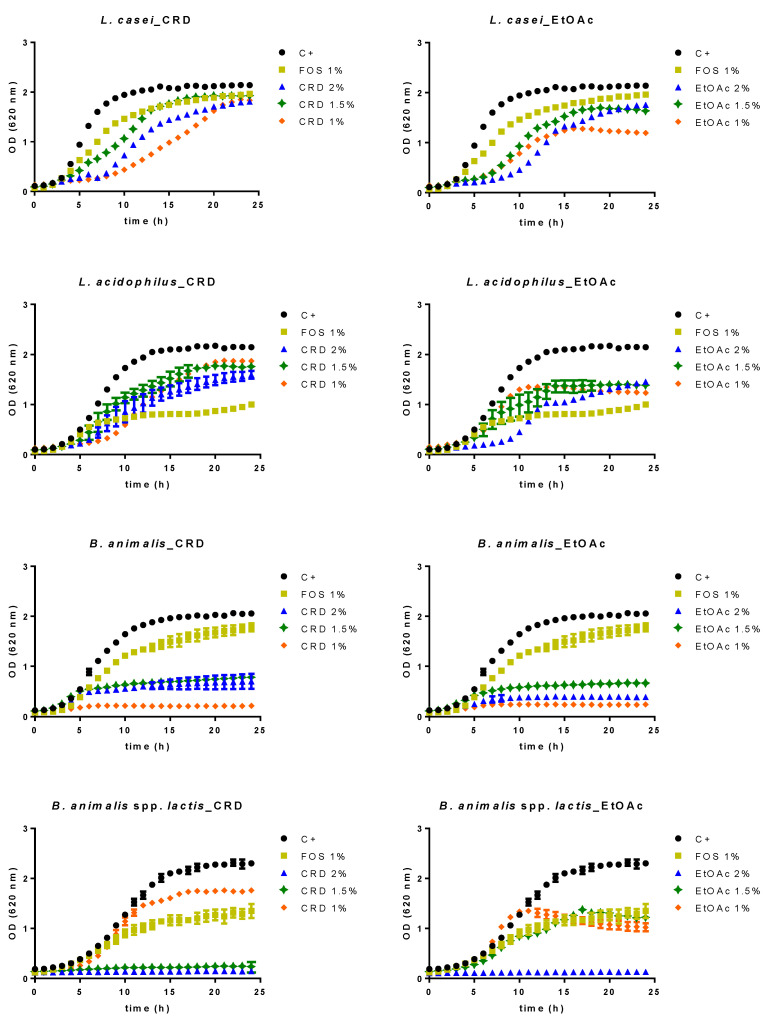
Growth curves of *L. casei*, *L. acidophilus*, *B. animalis* and *B. animalis* spp. *lactis* in the presence of different concentrations of digested crude extract (CRD) and ethyl acetate fraction (EtOAc). Data represent the mean ± SD of at least three independent assays.

The prebiotic activity of digested *F. vesiculosus* CRD and EtOAc was studied on four strains in basal Man–Rogosa–Sharpe (MRS) broth without glucose, at concentrations of 1–2% (*w*/*v*). Figure 1 presents the growth curves of the evaluated *Lactobacillus* and *Bifidobacteria* strains over 24 h, as no further alterations were observed between 24 and 48 h. All the probiotic microorganisms were affected by the presence of the *F. vesiculosus* samples in different manners. *Lactobacillus casei* exhibited a growth behavior identical for almost all the conditions tested, with no differences observed on the maximum optical density (OD), although the seaweed samples seemed to slightly delay their growth during the first 10 h. The only notable exception was EtOAc at 1%, which caused a slight decrease of the growth curve of this strain. In turn, the incubation of *L. acidophilus* with either CRD or EtOAc presented a growth curve considerably higher than that of FOS, for all the concentrations tested, thus indicating that both CRD and EtOAc stimulate the growth of this strain.

In contrast, *B. animalis* growth was the least pronounced of all the strains tested, in the presence of either CRD or EtOAc, suggesting that they might exert a bacteriostatic effect on this strain. The results for *B. animalis* spp. *lactis* demonstrated that the CRD at 1% displayed better stimulatory effects than FOS, although the bacterial growth was completely abolished for higher concentrations, indicating that, in such conditions, this extract impairs the growth of this strain. Positive stimulatory effects were noticed for EtOAc at 1 and 1.5% as well, which demonstrated growth curves identical to that of FOS. However, for the concentration of 2%, this sample also exhibited inhibitory effects toward this strain.

The potential prebiotic effect of seaweeds is a subject barely studied so far. Nevertheless, Martelli et al. [26] recently showed that four strains of probiotic bacteria (*L. casei*, *L. paracasei*, *L. rhamnosus* and *B. subtilis*) all exhibited good capacity to grow in a broth medium containing *Himanthalia elongata* flour (5%), which is in line with previous works that demonstrated the capacity of different brown algae species (*Sargassum siliquanstrum*, *Laminaria digitata*, *Laminaria saccharina*) to stimulate the growth of several probiotic bacteria including *Weissella* spp., *Lactobacillus* spp., *Leuconostoc* spp., *L. plantarum* and *L. rhamnosus* [27,28,29]. However, seaweeds have a very complex matrix and the contribution of phlorotannins for the effects observed by these authors are likely to be negligible. In fact, current knowledge regarding the fate of seaweed polyphenols in the human gastrointestinal tract is scarce. In the work developed by Corona et al. [30], after submitting a polyphenol-rich extract from *A. nodosum* to a simulated gastrointestinal digestion followed by fecal fermentation, they were able to find seven phlorotannin-derived metabolites, and, although the microbiota composition was not assessed, the presence of these metabolites suggests that phlorotannins might have been used by the colonic bacteria. In turn, in a 24 h in vitro fermentation carried out using *Ecklonia radiata* phlorotannin extract, a significant increase in the populations of Bacteroidetes, *Clostridium coccoides*, *E. coli* and *Faecalibacterium prausnitzii* was observed, although the levels of *Bifidobacterium* and *Lactobacillus* populations were found to be decreased [19]. With these results, we demonstrate for the first time that *F. vesiculosus* extract and phlorotannin-enriched fraction can stimulate the growth of some probiotic strains in a similar way to that of FOS.

### 2.3. Evolution of the Gut Microbiota Profile Groups

After GIT simulation, the digested *F. vesiculosus* CRD and EtOAc were submitted to human feces fermentation during 48 h, and aliquots were taken at 0, 12, 24 and 48 h to study their effect upon the human microbiota. Three of the four dominant phyla in the human gut were evaluated, namely Firmicutes (represented by *Clostridium leptum*, *Enterococcus* spp. and *Lactobacillus* spp.), Bacteroidetes (represented by *Bacteroides* spp.) and Actinobacteria (represented by *Bifidobacterium* spp.), and the compositional averages of the copy numbers obtained by real-time PCR of these main groups are depicted in the Table 2.

**Figure 2:**
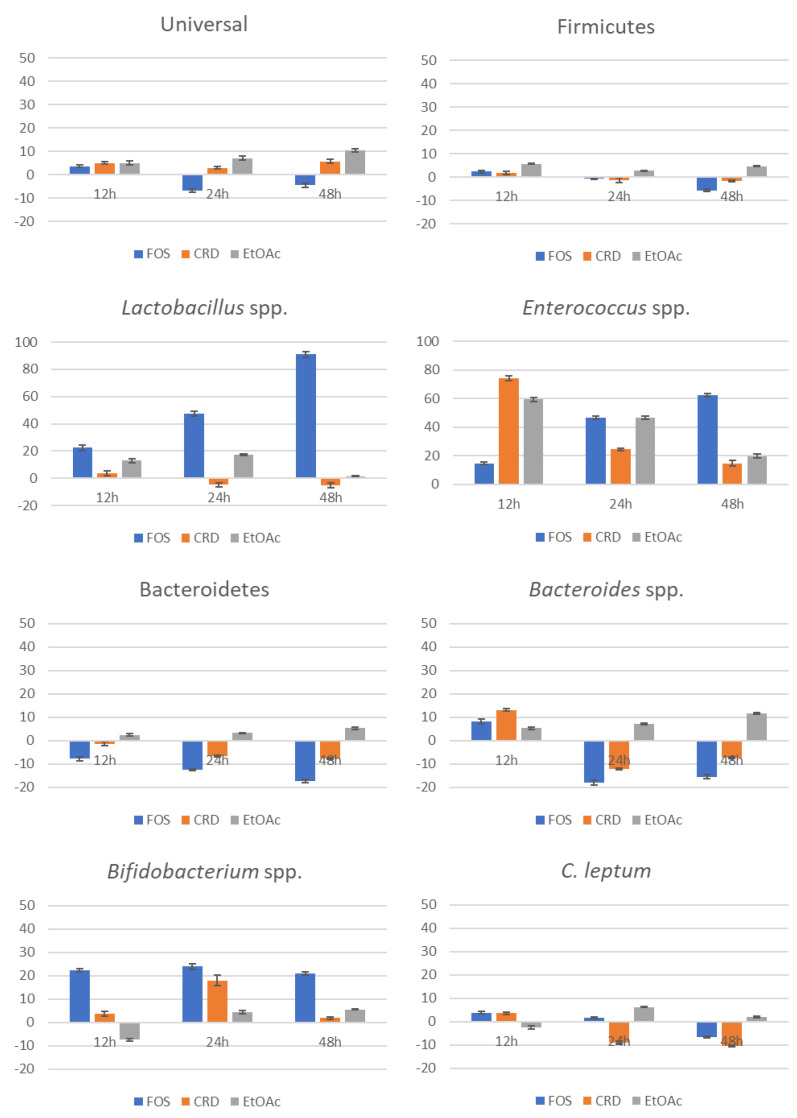
Evolution of the gut microbiota groups (relative differences to negative control in %) along the fermentation. Data represent the mean ± SD of five independent assays.

The numbers were in agreement with those found in healthy volunteers’ feces, with *Clostridium*, *Bacteroides* and *Bifidobacterium* comprising the dominant genera while *Lactobacillus* spp. and *Enterococcus* appeared as the subdominant genera [31,32,33]. Figure 2 depicts the relative differences (in %) between the microbiota groups of the tested samples and control feces, along 12, 24 and 48 h of fermentation. Overall, both CRD and EtOAc promoted a modest positive effect on gut microbiota growth, as noticed by the increment in the universal microorganisms compared to the control over time, while FOS exerted a positive effect on the initial 12 h that reversed for the following 24 and 48 h.

The EtOAc fraction caused a positive effect over time on the phyla Firmicutes and Bacteroidetes, which are representative of a healthy microbiota [34], while CRD and FOS exhibited a null or negative effect on these two groups. In turn, as expected, FOS exerted a very positive effect on *Lactobacillus* spp. and *Bifidobacterium* spp., two genera that are the markers of prebiosis par excellence. Likewise, despite not having an effect as sharp as FOS, EtOAc fraction also positively stimulated the growth of these two probiotic groups over time, although in the case of *Lactobacillus* spp., the effect lasted only until 24 h, becoming null at the end of the fermentation (48 h). Identical behavior was noticed for CRD on *Bifidobacterium* spp., promoting their growth only during the first 24 h. Curiously, no effect was observed on *Lactobacillus* spp., contrarily to what was expected since *L. casei* and *L. acidophilus* responded with a very positive growth behavior in the presence of this sample on the prebiotic studies (Section 2.2).

Interestingly, the group of *Enterococcus* spp. was the most beneficiated by CRD and EtOAc, although the levels of these organisms progressively decreased over time, contrarily to FOS which promoted their growth at each time point. Poor gut health outcomes have generally been linked to this genus [35], although this is a controversial subject since not all enterococcal strains cause health problems. In fact, strains such as *E. faecium* SF68^®^ and *E. faecalis* Symbio-flor^®^ have been marketed as probiotics for two decades without incidence and with very few reported adverse events [36]. Moreover, enterococcal probiotics have been shown to be effective in limiting gastrointestinal infectious burden and in the treatment of gastrointestinal infections and diarrhea [37].

On the contrary, *Clostridium leptum,* an important butyrate-producing strain, was the least affected by the studied samples, with only CRD causing a slight negative effect on its growth over time.

Regarding *Bacteroides* spp., the results demonstrated that even though all the samples promoted an increment in this group during the first 12 h, only EtOAc maintained this positive effect throughout the fermentation course. Instead, FOS and CRD turned out to negatively affect the growth of these bacteria after 24 h and until the end of the fermentation. Similar to *Enterococcus* spp., there is some controversy around the probiotic potential of the genus *Bacteroides*. On one hand, this group has been associated with the development of intestinal dysfunctions such as diarrhea, inflammatory bowel disease and colorectal cancer, and, on the other hand, it has been recently considered as a next generation probiotic candidate due to its potential role in promoting host health through the regulation of intestinal redox levels or the production of important short-chain fatty acids such as acetate, propionate and butyrate, which in turn can contribute to the regulation of toxin transport from the gut lumen to blood, the prevention of colon cancer and the prevention of inflammatory conditions [38].

**Figure 3:**
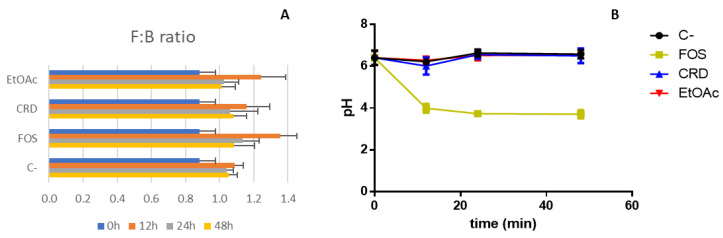
Firmicutes:Bacteroidetes (F:B) ratio (**A**) and variation of the pH (**B**) throughout the fermentation of digested FOS, CRD and EtOAC with human microbiota. Data represent the mean ± SD of five independent assays.

Another important aspect to consider is the ratio between Firmicutes and Bacteroidetes (F:B), the most predominant phyla in the human colon. Together they comprise 90% of the total gut microbiota and, thus, their proportion can give us a global idea of the total effect of *F. vesiculosus* samples on the intestinal flora. Commonly, healthy individuals display a nearly 1:1 ratio of Firmicutes to Bacteroidetes, and significant alterations of this ratio have been associated with pathological states [39]. For instance, increased F:B ratios have been linked to the pathophysiology of obesity [40], while patients of type II diabetes mellitus were found to have their levels of Firmicutes significantly reduced compared to their non-diabetic counterparts and consequently had decreased F:B ratios [41]. In this work, a slight increase of the F:B ratio was noticed for FOS and EtOAc (1.36 ± 0.10 and 1.24 ± 0.14, respectively) compared to the control (1.09 ± 0.05) during the first 12 h of fermentation, which then returned to normal levels over the next 24 and 48 h (Figure 3A). On the contrary, CRD did not cause any significant alterations of this parameter maintaining the F:B ratio values stable and close to one over the course of the fermentation.

Very few studies focusing on the prebiotic potential of phlorotannin-rich extracts have been conducted so far, although there are already some insights on this matter. Interestingly, Charoensiddhi et al. [19] reported that, after the 24 h fermentation period of a phlorotannin-rich extract of *E. radiata* with human fecal samples, only the group of Bacteroidetes showed an increased growth compared to the negative control, while Firmicutes and *Bifidobacterium* spp. remained unchanged and *Lactobacillus* spp. and *Enterococcus* spp. actually decreased. However, these authors also observed a stimulation of the growth of *Faecalibacterium prausnitzii* and *Clostridium coccoides*, which were not analyzed in this study but are two important groups associated to SCFA production (particularly butyrate) and health-promoting effects [42,43]. In a different work, the administration of a polyphenol-rich extract from the brown algae *Lessonia trabeculata* to streptozotocin-induced diabetic rats under a high-fat diet significantly restored the levels of the three dominant phyla, i.e., Firmicutes, Bacteroidetes and Proteobacteria, as well as the F:B ratio to values identical of the negative control [20]. To the authors knowledge, this work was the first assessing the potential modulatory effects of *F. vesiculosus* phlorotannin extracts on human gut microbiota and allowed the disclosure of valuable information on how *F. vesiculosus* phlorotannins may impact on the human gastrointestinal microflora.

### 2.4. Organic Acids Profile and pH Variation

The changes in the concentration of short-chain fatty acids along the fermentation of FOS, CRD and EtOAc with human feces in basal media were analyzed by HPLC and are presented in Table 3. SCFAs such as acetate, propionate and butyrate are volatile fatty acids that are produced by the gut microbiota in the colon as a result of the fermentation and metabolization of food components that are undigested/unabsorbed in the upper GIT.

In this study, fermentation with FOS caused a remarkable increase in the production of total organic acids, while in the fermentations carried out with *F. vesiculosus* samples, a tendential increase in the total organic acid levels was noticed despite not being statistically significant when compared with the negative control. These results are also reflected in the pH changes registered during the fermentation (Figure 3B), with FOS producing a significant decrease in the pH values, while the pH registered for CRD and EtOAc remained similar to that of the control, at least for the time window tested. Differences in the SCFA profiles, however, were detected between samples. One of the most evident differences was noticed for lactate, which was the main metabolite produced over the entire fermentation of FOS. This outcome also relates with the high stimulatory effects that FOS produced on *Lactobacillus* spp. and *Bifidobacterium* spp. validated above with the 16S rRNA gene analysis (Section 2.3). On the other hand, similar to the negative control, the lactate production in fermentations carried out with CRD and EtOAc were nearly null, and even undetectable, at 48 h. An identical pattern was found for acetate production, which was remarkably stimulated in the presence of FOS but not affected by CRD or EtOAc. Under normal conditions this acid together with propionic and butyric acids comprise the three major SCFAs normally produced in the gut, which are important for the maintenance of intestinal homeostasis [44]. In particular, acetate plays a very important role in energy homeostasis, contributing to appetite regulation, promoting fat oxidation, improving insulin sensitivity and glucose homeostasis, and enhancing the inflammatory status [45].

Interestingly, all three samples promoted an increase in the succinate level, which reached its maximum at 12 h and was kept constant for FOS until the end of the fermentation, while for CRD and EtOAc it decreased over time. On one hand, the accumulation of this organic acid in the gut lumen is usually associated with microbiota disturbances commonly linked to poor gut health states such as antibiotic-induced dysbiosis, motility disturbances and specifically IBD [46]. On the other hand, succinate is also a key intermediate in the production of propionate, which in turn is responsible for modulating lipogenesis, controlling appetite and preventing colon cancer [47]. In fact, the levels of propionate production herein noted seem to follow an identical behavior compared with that of succinate, showing an accentuated increase during the initial phase of the fermentation and a decrease at the end, only for CRD and EtOAc. Indeed, high correlation coefficients between these two organic acids were obtained (R^2^ = 0.99, 0.88 and 0.97 for FOS, CRD and EtOAc, respectively), which confirms that the production of propionate is indeed associated with the production of succinate.

One of the most important SCFAs produced in the gut is butyrate, which has been repeatedly reported for its positive health-promoting effects. In addition to its function as the primary energy source for colonocytes, butyrate also importantly contributes to the improvement of the gut barrier function, exerts anti-inflammatory and regenerative effects, prevents the formation of colon cancer and helps reduce both type II diabetes and obesity [39]. Therefore, stimulating the production of high levels of this SCFA is of great interest for promoting the healthy function of the gut. The results herein obtained revealed that only the EtOAc led to a significant increase in butyric acid, while, in CRD and FOS, the levels of this SCFA did not differ much from the negative control.

The main butyrate-producing bacteria are *Faecalibacterium prausnitzii*, *Clostridium* spp., *Eubacterium* spp., *Roseburia* spp. and *Anaerostipes* spp., which belong to the Firmicutes phylum [44], and, despite the fact that the *C. leptum* did not show significant positive growth in the presence of EtOAc, an increase in the group Firmicutes was noticed on the EtOAc-fermented samples, which might explain the increased levels of butyrate registered. In turn, the lack of production of butyrate, which was not expected, in the FOS fermentation could be possibly explained by the absence of the common cross-feeding effect among intestinal bacteria that produce acetate, propionate or butyrate as the final product of lactate metabolization [48]. Indeed, the fact that lactate has accumulated so much throughout the fermentation of FOS indicates that it has not been utilized as a substrate by other bacteria. Nevertheless, it must be considered that this experiment was performed without pH control, and, thus, it is likely that the sharp decrease in the pH may have impaired the growth of certain lactate-utilizing bacteria and favored the growth of the lactate-producing ones, therefore contributing to the increasing accumulation of this organic acid at the expense of other SCFAs [49].

When comparing these results with those previously reported by Charoensiddhi et al. [19], the stimulatory effects of *F. vesiculosus* phlorotannin samples herein tested on the production of SCFAs were much more promising than those of the *E. radiata* phlorotannin-rich extract used in their study. In fact, the authors reported that the fermentation of the phlorotannin extract caused a reduction on the levels of total SCFAs with a remarkable decrease in the concentration of acetic acid in comparison with the negative control. Contrarily, Yuan et al. [20] found that the administration of a polyphenol-rich extract from the brown algae *Lessonia trabeculata* to streptozotocin-induced diabetic rats, under a high-fat diet, significantly restored the levels of acetate and butyrate that were depleted in the diabetic control groups. Notably, the levels of butyrate in the treated rats were even higher than those of the control group, i.e., healthy rats. In our study, despite the fact that the total SCFA production was much lower than that observed for FOS, both CRD and EtOAc exhibited interesting alterations in the SCFA profiles stimulating the production of propionate and, in the case of EtOAc, butyrate as well. These SCFAs could exert interesting beneficial health properties not only in the colon and gut microbiota but also in other organs, which could partly explain the health benefits attributed to phlorotannins.

## 3. Materials and Methods

### 3.1. Chemicals

Grounded *Fucus vesiculosus* harvested in July 2017 was purchased from Algaplus Lda. Acetone, methanol, *n*-hexane, ethyl acetate, DMSO, glacial acetic acid, hydrochloric acid and sodium hydroxide were acquired from Fisher (Pittsburgh, PA, USA). Sodium nitroprusside and sulfanilamide were ordered from Acros Organics (Hampton, NH, USA). Ascorbic acid, gallic acid, NADH, NBT, PMS, FOS, DMBA, α-amylase, paraffin, bile salts, pancreatin, pepsin, sodium hydrogen carbonate, D-glucose, organic acids (succinate, lactate, propionate, butyrate and acetate) and sulfuric acid were obtained from Sigma (St. Louis, MO, USA). Man–Rogosa–Sharpe (MRS) medium and L-cysteine-HCl were purchased from Biokar (Allonne, France) and Merck (Darmstadt, Germany), respectively, while trypticase soya broth (TSB) without dextrose and bactopeptone were acquired from BBL (Cockeysville, Maryland, MD, USA) and Amersham (Buckinghamshire, UK), respectively. Salt solution A (100.0 g/L NH_4_Cl, 10.0 g/L MgCl_2_·6H_2_O, 10.0 g/L CaCl_2_·2H_2_O), salt solution B (200.0 g/L K_2_HPO_4_·3H_2_O) and resazurin solution were ordered from ATCC (Manassas, VA, USA). Sodium di-hydrogen phosphate and potassium di-hydrogen phosphate were purchased from Panreac (Barcelona, Spain). Dinitrosalicylic acid and acarbose were purchased from Acros Organics (Hampton, NH, USA), calcium chloride from ChemLab (Eernegem, Belgium) and orlistat from AlfaAesar (Ward Hill, MA, USA). Finally, the *Bifidobacterium animalis* BB0 were acquired from CSK (Ede, The Netherlands), *Bifidobacterium animalis* spp. *lactis* Bb12 and *Lactobacillus casei* 01 from Chr. Hansen (Hørsholm, Denmark) and *Lactobacillus acidophilus* La-5 from Lallemand (MontReal, QC, Canada).

### 3.2. Extraction Procedure

The extracts were prepared following the optimal conditions determined through the response surface method as previously described [50]. For this, 30 g of dried algal powder was dispersed in 2100 mL of 70% acetone solution and incubated for 3 h at room temperature under constant agitation. The mixture was filtered through cotton to remove the solid residues and then through a G4 glass filter. Afterward the extract was concentrated in a rotary evaporator to about 250 mL. The concentrated extract was defatted using *n*-hexane (1:1, *v*/*v*) for several times (until a colorless non-polar fraction was obtained), and the aqueous phase was further submitted to liquid–liquid extraction with ethyl acetate (1:1, *v*/*v*) for three times to obtain a phlorotannin-purified fraction (EtOAc). Finally, the solvent was removed from the EtOAc fraction by rotary evaporation. Both CRD and EtOAc were then freeze dried and stored at −20 °C until further use.

### 3.3. Gastrointestinal Digestion Simulation

The simulation of the gastrointestinal digestion of the *F. vesiculosus* sample extracts was performed according to the method described by Campos et al. [33]. Oral digestion was started by suspending 1 g of dried sample (CRD or EtOAc) in 20 mL of distilled water followed by the adjustment of the pH between 5.6 and 6.9 with NaHCO_3_ prior to the addition of 0.6 mL/min of α-amylase at 100 U/mL. Enzymatic digestion was carried out during 2 min of mastication, at 37 °C and 200 rpm. Before moving to the next compartment, the pH of mouth digest was adjusted to 2.0 using 1M HCl and then mixed with a simulated gastric juice consisting of pepsin 25 mg/mL added at a ratio of 0.05 mL/mL of mouth digest. Incubation was carried out over 60 min at 37 °C and 130 rpm. Finally, for intestinal digestion the pH of gastric digest was adjusted to 6.0 using 1M NaHCO_3_ prior to the addition of a simulated intestinal juice consisting of 2 g/L of pancreatin and 12 g/L bile salts at a ratio of 0.25 mL/mL of gastric digest. The samples were then incubated during 120 min, at 37 °C and 45 rpm, to mimic a long intestine digestion process. In the final step of intestinal digestion, samples were submitted to a dialysis process during 48 h at room temperature using a membrane with a molecular pore size of 3 kDa to reproduce the natural absorption step in the small intestine. At the end of this process, the permeate represented the bioaccessible fraction, while the retentate represented the non-absorbable fraction, both of which were then used for the fermentation experiments. An aliquot of 2 mL was collected before the digestion simulation and after each step of digestion, i.e., mouth digest, gastric digest, intestinal digest, permeate and retentate, and stored at −80 °C until further use for phlorotannin quantification and antioxidant experiments.

### 3.4. Determination of the Phlorotannin Content and Antioxidant Activities

Quantification of the TPhC was carried out according to the 2,4-dimethoxybenzaldehyde (DMBA) colorimetric method [51]. For this, equal volumes of the stock solutions of DMBA (2%, m/v) and HCl (6%, *v*/*v*), both prepared in glacial acetic acid, were mixed prior to use (work solution). Afterwards, 250 µL of this solution was added to 50 µL of each extract in a 96-well plate and the reaction was incubated in the dark, at room temperature. After 60 min, the absorbance was read at 515 nm and the phlorotannin content was determined by using a regression equation of the phloroglucinol linear calibration curve (0.06–0.1 mg/mL). The results were expressed as mg phloroglucinol equivalents/g dry seaweed (mg PGE/g DW).

The NO^●^ scavenging method was adapted from Pereira et al. [52]. For this, 100 µL of six different sample concentrations (0–1 mg/mL) was mixed with 100 µL of sodium nitroprusside (3.33 mM in 100 mM sodium phosphate buffer pH 7.4) and incubated for 15 min under a fluorescent lamp (Tryun 26 W). Next, 100 µL of Griess reagent (0.5% sulfanilamide and 0.05% *N*-(1-naphthyl)ethylenediamine dihydrochloride in 2.5% H_3_PO_4_) was added to the mixture, which was incubated for another 10 min at RT in the dark. The absorbance was then measured at 562 nm, and the NO^●^ scavenging capacity was calculated as the concentration of the sample capable of scavenging 50% of the radical. Ascorbic acid was used as the reference compound.

The O_2_^●–^ scavenging method was carried out according to the method described by Pereira et al. [53]. In a 96-well plate, 75 µL of six different sample concentrations (0.0–2.0 mg/mL) was mixed with 100 µL of β-NADH (300 µM), 75 µL of NBT (200 µM) and 50 µL of PMS (15 µM). After 5 min, the absorbances at 560 nm were recorded and the inhibition calculated as the concentration capable of scavenging 50% of O_2_^●−^ (IC_50_). Gallic acid was used as the reference compound.

### 3.5. Determination of the Phlorotannin Content and Antioxidant Activities

Potential prebiotic effects of *F. vesiculosus* phlorotannin-rich samples were determined for *Bifidobacterium animalis* B0, *Bifidobacterium animalis* spp. *lactis* BB12, *Lactobacillus casei* 01 and *Lactobacillus acidophilus* LA-5. Strains were stored at −80 °C in MRS broth with 30% (*v*/*v*) glycerol. *L. casei* 01 and *L. acidophilus* LA-5 inocula were prepared by suspending each bacterial colony into MRS broth, achieving a turbidity equivalent to 0.5 McFarland standard, and then diluting to reach the recommended concentration of probiotic bacteria in the wells, 5 × 10^5^ CFU/mL. Twenty microliters of each inoculum were transferred to a 96-well microplate and every well was fulfilled (to the final volume of 200 µL) with each *F. vesiculosus* sample, diluted in basal MRS broth without glucose at concentrations of 1, 1.5 and 2% (*w*/*v*). The microplate was incubated at 37 °C for 48 h with agitation. Similarly, *B. animalis* B0 and *B. lactis* BB12 inocula were prepared under an anaerobic atmosphere, by suspending each bacterial colony into MRS broth supplemented with 0.05% (*v*/*v*) L-cysteine-HCl, achieving a final turbidity equivalent to 0.5 McFarland standard, and then diluted to reach the recommended concentration of probiotic bacteria in the wells, 5 × 10^5^ CFU/mL. Twenty microliters of each inoculum were transferred to a 96-well microplate and every well was fulfilled (to the final volume of 200 µL) with each *F. vesiculosus* sample, diluted in basal MRS broth without glucose at concentrations of 1, 1.5 and 2% (*w*/*v*). The microplate was sealed with paraffin and incubated at 37 °C for 48 h with agitation. In all plates, OD measurements at 620 nm were registered every hour. Three controls were also performed: the first one containing inoculum and MRS broth with glucose (positive control), the second one containing inoculum and FOS in MRS broth without glucose (FOS control) and the third one containing only inoculum and MRS broth (negative control).

### 3.6. In Vitro Fermentation Assays

The human feces were collected into sterile plastic vases and kept under anaerobic conditions, until further notice (maximum of 2 h after collection). The samples were obtained fresh, from healthy human donors, with the premises of not having any known metabolic or gastrointestinal disorder. Moreover, the donors confirmed that they were not taking any probiotic or prebiotic supplements, as well as any form of antibiotics for the previous 3 months. The basal medium was prepared as described previously [33], consisting of a nutrient base medium containing 5.0 g/L trypticase soya broth (TSB) without dextrose (BBL, Cockeysville, Maryland, MD, USA), 5.0 g/L bactopeptone (Amersham, Buckinghamshire, UK), 0.5 g/L L-cysteine-HCl (Merck, Germany), 1.0% (*v*/*v*) of salt solution A (100.0 g/L NH_4_Cl, 10.0 g/L MgCl_2_6H_2_O, 10.0 g/L CaCl_2_2H_2_O), 0.2% (*v*/*v*) of salt solution B (200.0 g/L K_2_HPO_4_3H_2_O) and 0.2% (*v*/*v*) of 0.5 g/L resazurin solution, prepared in distilled water and with pH adjustment at 6.8. The basal medium was dispensed into airtight glass anaerobic bottles, sealed with aluminum caps before sterilization by autoclave. Stock solutions of yeast nitrogen base (YNB) were sterilized with 0.2 μm syringe filters (Chromafils, Macherey-Nagel, Düren, Germany) and inserted into the bottles. The serum bottles were incorporated with CRD and EtOAc extract retentate from the in vitro GIT simulation at a final concentration of 2% (*w*/*v*) and inoculated with fecal slurries of 2% (*v*/*v*) at 37 °C for 48 h without shaking nor pH control. Samples were taken at 0, 12, 24 and 48 h of fermentation. All the experiments were carried out inside an anaerobic cabinet with 5% of H_2_, 10% of CO_2_ and 85% of N_2_ and performed in compliance with the institutional guidelines.

### 3.7. Gut Microbiota Evaluation

#### 3.7.1. DNA Extraction

Genomic DNA was extracted and purified from stool samples as previously described [33] using NZY Tissue gDNA Isolation Kit (Nzytech, Lisbon, Portugal) with some modifications. Samples were centrifuged at 11,000 *g* during 10 min to separate the supernatant from the pellet. Around 170–200 mg of pellet was taken from the control and test samples for all times. After, the pellets were homogenized in TE buffer (10 mM Tris/HCl; 1 mM EDTA, pH 8.0) and centrifuged again at 4000 *g* for 15 min. The supernatant was discarded, and the pellet was resuspended in 350 μL of buffer NT1. After an incubation step at 95 °C for 10 min, the samples were centrifuged at 11,000 *g* for 1 min. Then, 25 μL of proteinase K was added to 200 μL of supernatant and incubated at 70 °C for 10 min. The remaining steps followed the manufacturer’s instructions. The DNA purity and quantification were assessed with a NanoDrop spectrophotometer (ThermoScientific, Wilmington, DE, USA).

#### 3.7.2. Real-Time PCR for Microbial Analysis of Stool

Real-time PCR was performed as described before in [33] in sealed 96-well microplates using a LightCycler FastStart DNA Master SYBR Green kit and a LightCycler instrument (Roche Applied Science, Indianapolis, ID, USA). PCR reaction mixtures (total of 10 μL) contained 5 μL of 2 × Faststart SYBRGreen (Roche Diagnostics Ltd., Burgess Hill, UK), 0.2 μL of each primer (final concentration of 0.2 μM), 3.6 μL of water and 1 μL of DNA (equilibrated to 20 mg). Primer sequences (Sigma-Aldrich, St. Louis, MO, USA) used to target the 16S rRNA gene of the bacteria and the conditions for PCR amplification reactions are reported in Table 4.

To verify the specificity of the amplicon, a melting curve analysis was performed via monitoring SYBR Green fluorescence in the temperature ramp from 60 to 97 °C. Data were processed and analyzed using the LightCycler software (Roche Applied Science, Penzberg, Germany). Standard curves were constructed using serial tenfold dilutions of bacterial genomic DNA, according to the following webpage http://cels.uri.edu/gsc/cbdna.html (accessed at 31 March 2021). Bacterial genomic DNA used as a standard (Table 4) was obtained from DSMZ (Braunschweig, Germany). Genome size and the copy number of the 16S rRNA gene for each bacterial strain used as a standard was obtained from the NCBI Genome database (http://www.ncbi.nlm.nih.gov, accessed at 31 March 2021). Data are presented as the mean values of duplicate PCR analyses. The F:B ratio was obtained by dividing the number of copies of Firmicutes divisions by the number of copies of Bacteroidetes divisions. Moreover, the relative differences to negative control percentage (only feces fermentation) were calculated using the following equation:

$$Relative\;difference\;to\;control\;\%=\frac{SMC-CMC}{CMC}\times 100$$

where *SMC* is the mean copy number of the sample at a certain time (12, 24 or 48 h) and *CMC* is the mean copy number of the control sample at the same time as *SMC*. Positive % values mean the occurrence of an increase in the number of copies relative to the control sample at that certain time. The higher the value, the higher increase.

#### 3.7.3. Determination of Organic Acids

Supernatants from the batch cultures were filtered through 0.2 μm cellulose acetate membranes. The chromatographic analysis was performed using a Beckman & Coulter 168 series HPLC system with refractive index-RI detector (Knauer, Berlin, Germany). The separation was performed using Aminex HPX-87H column (BioRad, Hercules, CA, USA) operated at 50 °C; mobile phase, 0.003 mol/L H_2_SO_4_; flow, 0.6 mL/min. Aliquots of the filtered samples were assayed for organic acids (lactic, acetic, succinic, propionic and butyric) using an Agilent 1200 series HPLC system with an RI detector (Agilent, Germany) and a UV detector.

### 3.8. Statistical Analysis

Data are expressed as mean ± SD of three similar and independent experiments and analyzed using a one-way ANOVA followed by Tukey’s post hoc test. The statistical tests were applied using GraphPad Prism, version 7.00 (GraphPad Software, San Diego, CA, USA) and the significance level was *p* < 0.05.

## 4. Conclusions

Overall, this work provides a great contribution for the understanding of the stability of the phlorotannins of *F. vesiculosus* along the digestive tract, as well as their bioaccessibility and stimulatory effects toward gut microbiota and SCFA production. Similar to plant polyphenols, phlorotannins seem to be susceptible to gut environmental conditions leading to a decrease in their concentration and antioxidant activity along the digestive tract. Moreover, from the portion of phlorotannins that can reach the intestinal lumen intact, only a small fraction of less than 15% will become bioaccessible and available for absorption, which indicates that the majority of these compounds will accumulate in the large intestine where they will be exposed to the metabolic activity of the gut microbiota. Meanwhile, the fermentation of the digested CRD and EtOAc revealed a slight positive effect on the growth of certain commensal bacteria from the human gut, with *Enterococcus* spp. showing the most relevant growth. Moreover, both samples demonstrated an interesting capacity to enhance the production of propionate, while EtOAc caused a notable increase in butyrate levels, both representing important short-chain fatty acids known for their health-promoting status.

In summary, the data gathered herein provide valuable information regarding the behavior of *F. vesiculosus* phlorotannins along their passage through the gastrointestinal tract, and even though the results obtained do not allow to claim *F. vesiculosus* phlorotannin extracts as prebiotics they present clear evidence that these compounds can still positively contribute to the maintenance of a healthy gastrointestinal condition. From here, it would be important to address whether fermentation with human colonic bacteria could affect the antioxidant and other bioactive properties of *F. vesiculosus* CRD and EtOAc. Moreover, it would be particularly relevant to disclose the possible formation of phlorotannin metabolites resultant from the biotransformation and bacterial metabolization in the colon.

## Figures and Tables

**Table 1:** Total phlorotannin content and antioxidant activity of *F. vesiculosus* crude and ethyl acetate fraction through the different stages of gastrointestinal digestion.

Sample	GIT Stage	TPhC (mg PGE/g ext)	^(1)^ NO^●^ (IC_50_ μg/mL)	^(2)^ O_2_^●–^ (IC_50_ μg/mL)
CRD	Undigested	9.93 ± 1.48 ^a^	161 ± 8.8 ^a^	417 ± 164.5 ^a^
	Mouth	6.33 ± 2.96 ^b^	309 ± 105.2 ^b^	745 ± 88.2 ^b^
	Stomach	8.52 ± 1.16 ^a^^,^^b^	171 ± 27.1 ^a^	378 ± 26.6 ^a^
	Intestine	5.17 ± 0.70 ^b^	287 ± 27.2 ^a^^,^^b^	1105 ± 421.3 ^b^
	Retentate *	4.60 ± 0.26 ^b^	141 ± 9.1 ^a^	294 ± 19.3 ^a^
	Permeate *	1.40 ± 0.19 ^c^	2551 ± 30.7 ^c^	2580 ± 75.2 ^c^
EtOAc	Undigested	17.39 ± 1.77 ^a^	45 ± 2.5 ^a^	118 ± 17.6 ^a^
	Mouth	13.83 ± 0.74 ^b^	73 ± 11.0 ^a^^,^^b^	221 ± 1.1 ^a^^,^^b^
	Stomach	5.67 ± 0.91 ^c^	109 ± 7.1 ^a^^,^^b^	244 ± 0.4 ^a^^,^^b^
	Intestine	3.28 ± 0.55 ^c^	195 ± 38.5 ^b^^,^^c^	564 ± 19.9 ^c^
	Retentate *	2.97 ± 0.62 ^c^^,^^d^	281 ±16.1 ^c^	383 ± 18.2 ^b^^,^^c^
	Permeate *	0.37 ± 0.10 ^d^	1531 ± 52.2 ^d^	3074 ± 32.3 ^d^
Standard compound		-	36 ± 0.9	6 ± 0.5

CRD—crude extract; EtOAc—ethyl acetate fraction; GIT—gastrointestinal tract; TPhC—total phlorotannin content. ^(1)^ Standard compound for NO^•^ is ascorbic acid; ^(2)^ standard compound for O_2_^•−^ is gallic acid; * results for DMBA expressed in mg PGE/g intestine digest. Data represent the mean ± SD of at least three independent assays. For each sample, different letters indicate significant differences within the same column (*p* < 0.05).

**Table 2:** Fecal microbiota composition of volunteer participants.

Division (Genus)	Number of Copies (n = 5) ^a^
Universal	7.52 ± 0.38
Firmicutes	4.76 ± 0.20
*Clostridium leptum*	4.97 ± 0.26
*Enterococcus* spp.	2.07 ± 0.63
*Lactobacillus* spp.	3.27 ± 0.72
Bacteroidetes	5.46 ± 0.63
*Bacteroides* spp.	3.76 ± 0.55
*Bifidobacterium* spp.	4.42 ± 0.45
F:B ratio	0.97 ± 0.23

^a^ Values are presented as mean ± SD of five independent assays and expressed as log10 16S rRNA gene copies per 20 ng of DNA.

**Table 3:** Concentration of organic acids (succinic, lactic, acetic, propionic and butyric) throughout fermentation of digested FOS, CRD and EtOAC with human microbiota (mg/mL).

Organic Acids	Time (h)	Ctrl	FOS	CRD	EtOAc
Total	0	2.38 ± 0.63 ^a;A^	2.38 ± 0.63 ^a;A^	2.38 ± 0.63 ^a;A^	2.38 ± 0.63 ^a;A^
	12	5.24 ± 1.98 ^a;A^	10.89 ± 2.79 ^b;B^	7.43 ± 2.09 ^b;A^	7.76 ± 1.92 ^b;A,B^
	24	4.90 ± 1.59 ^a;A^	12.63 ± 2.37 ^b,c;B^	7.08 ± 2.45 ^b;A^	7.55 ± 1.75 ^b;A^
	48	4.10 ± 2.01 ^a;A^	14.78 ± 4.00 ^c;B^	5.04 ± 1.57 ^a,b;A^	6.38 ± 1.98 ^b;A^
Succinic acid	0	0.45 ± 0.20 ^a;A^	0.45 ± 0.20 ^a;A^	0.45 ± 0.20 ^a;A^	0.45 ± 0.20 ^a;A^
	12	0.77 ± 0.75 ^a;A^	1.85 ± 0.92 ^b;B^	2.29 ± 1.39 ^c;B^	2.15 ± 1.15 ^b;B^
	24	1.12 ± 0.53 ^a;A^	1.97 ± 0.58 ^b;B^	2.02 ± 0.93 ^b,c;A,B^	1.35 ± 0.50 ^a,b;A,B^
	48	0.74 ± 0.71 ^a;A^	2.03 ± 0.85 ^b;B^	1.12 ± 0.20 ^a,b;A,B^	1.40 ± 0.89 ^a,b;A,B^
Lactic acid	0	ND	ND	ND	ND
	12	1.21 ± 0.93 ^a;A^	3.91 ± 1.94 ^a;B^	0.87 ± 0.22 ^a;A^	0.87 ± 0.23 ^a;A^
	24	0.34 ± 0.14 ^a;A^	4.81 ± 0.75 ^a,b;B^	0.76 ± 0.58 ^a;A^	0.26 ± 0.16 ^a;A^
	48	ND	5.49 ± 2.14 ^b^	ND	ND
Acetic acid	0	0.16 ± 0.04 ^a;A^	0.16 ± 0.04 ^a;A^	0.16 ± 0.04 ^a;A^	0.16 ± 0.04 ^a;A^
	12	0.81 ± 0.10 ^b;A^	1.36 ± 0.75 ^b;A^	1.03 ±0.09 ^b;A^	1.02 ± 0.19 ^b;A^
	24	0.82 ± 0.17 ^b;A^	1.65 ± 0.52 ^b;B^	0.92 ± 0.22 ^b;A^	0.96 ± 0.30 ^b;A^
	48	0.78 ± 0.20 ^b;A^	2.77 ± 1.21 ^c;B^	0.78 ± 0.20 ^b;A^	0.93 ± 0.28 ^b;A^
Propionic acid	0	0.34 ± 0.09 ^a;A^	0.34 ± 0.09 ^a;A^	0.34 ± 0.09 ^a;A^	0.34 ± 0.09 ^a;A^
	12	0.53 ± 0.23 ^a;A^	1.48 ± 0.32 ^b;B^	1.14 ± 0.49 ^b;B^	1.43 ± 0.87 ^b;B^
	24	0.65 ± 0.35 ^a;A^	1.89 ± 0.75 ^b;C^	1.25 ± 0.58 ^b;B^	0.85 ± 0.27 ^a,b;A,B^
	48	0.50 ± 0.24 ^a;A^	1.64 ± 0.60 ^b;B^	0.77 ± 0.20 ^a,b;A^	0.90 ± 0.24 ^a,b;A^
Butyric acid	0	1.41 ± 0.25 ^a;A^	1.41 ± 0.25 ^a;A^	1.41 ± 0.25 ^a;A^	1.41 ± 0.25 ^a;A^
	12	1.92 ± 0.69 ^a;A^	2.29 ± 0.99 ^a;A^	2.10 ± 0.79 ^a;A^	2.71 ± 0.94 ^a;A^
	24	2.24 ± 0.67 ^a;A^	2.23 ± 0.86 ^a;A^	2.54 ± 1.05 ^a;A^	4.12 ± 0.37 ^b;B^
	48	2.23 ± 1.35 ^a;A^	2.70 ± 1.43 ^a;A^	2.31 ± 0.85 ^a;A^	4.31 ± 0.62 ^b;B^

Ctrl—negative control; FOS—fructo-oligosaccharides; CRD—crude extract; EtOAc—ethyl acetate fraction; ND—not detected. Different letters indicate significant differences (*p* < 0.05). The capital letters indicate the differences among the Ctrl, FOS, CRD and EtOAc for organic acid concentration at the same time (same row), and the lowercase letters indicate the differences for the same sample over time for each organic acid concentration (same column within an organic acid). Data represent the mean ± SD of five independent assays.

**Table 4:** Primer sequences and real-time PCR conditions used for gut microbiota analysis.

Target Group	Maximum Growth Rate (µmax.h^−1^)			
	Primer Sequence (5′–3′)	Genomic DNA Standard	PCR Product Size (bp)	AT (°C)
Universal	AAA CTC AAA GGA ATT GAC GG ACT TCA CGA GCT GAC	*Bacteroides vulgatus* ATCC 8482 (DSMZ 1447)	180	45
Firmicutes	ATG TGG TTT AAT TCG AAG CA AGC TGA CGA CAA CCA TGC AC	*Lactobacillus gasseri* ATCC 33323 (DSMZ 20243)	126	45
*Enterococcus* spp.	CCC TTA TTG TTA GTT GCC ATC ATT ACT CGT TGT ACT TCC CT TGT	*Enterococcus gilvus* ATCC BAA-350 (DSMZ 15689)	144	45
*Lactobacillus* spp.	GAG GCA GCA GTA GGG AAT CTT C GGC CAG TTA CTA CCT CTA TCC TTC TTC	*Lactobacillus gasseri* ATCC 33323 (DSMZ 20243)	126	55
Bacteroidetes	CAT GTG GTT TAA TTC GAT GAT AGC TGA CGA CAA CCA TGC AG	*Bacteroides vulgatus* ATCC 8482 (DSMZ 1447)	126	45
*Bacteroides* spp.	ATA GCC TTT CGA AAG RAA GAT CCA GTA TCA ACT GCA ATT TTA	*Bacteroides vulgatus* ATCC 8482 (DSMZ 1447)	495	45
*Bifidobacterium* spp.	CGC GTC TGG TGT GAA AG CCC CAC ATC CAG CAT CCA	*Bifidobacterium longum* subsp. *infantis* ATCC 15697 (DSMZ 20088)	244	50

AT—annealing temperature; bp—base pairs; PCR—polymerase chain reaction.

## Data Availability

Data are available from the corresponding author.
